# Differential expression of pathogenicity- and virulence-related genes of *Xanthomonas axonopodis* pv. citri under copper stress

**DOI:** 10.1590/S1415-47572010005000030

**Published:** 2010-06-01

**Authors:** Ana Carolina Basílio Palmieri, Alexandre Morais do Amaral, Rafael Augusto Homem, Marcos Antonio Machado

**Affiliations:** 1 Centro APTA Citros Sylvio Moreira, Instituto Agronômico de Campinas, Cordeirópolis, SPBrazil; 2 , Embrapa Recursos Genéticos e Biotecnologia Brasília, DFBrazil; 3 Instituto de Biologia, Universidade Estadual de Campinas, Campinas, SPBrazil

**Keywords:** copper resistance, citrus canker, real-time quantitative PCR

## Abstract

In this study, we used real-time quantitative PCR (RT-qPCR) to evaluate the expression of 32 genes of *Xanthomonas axonopodis* pv. citri related to pathogenicity and virulence that are also involved in copper detoxification. Nearly all of the genes were up-regulated, including *cop*A and *cop*B. Two genes homologous to members of the type II secretion system (*xcs*H and *xcs*C) and two involved in the degradation of plant cell wall components (*pgl*A and *pel*) were the most expressed in response to an elevated copper concentration. The type II secretion system (*xcs* operon) and a few homologues of proteins putatively secreted by this system showed enhanced expression when the bacteria were exposed to a high concentration of copper sulfate. The enhanced expression of the genes of secretion II system during copper stress suggests that this pathway may have an important role in the adaptative response of *X. axonopodis* pv. citri to toxic compounds. These findings highlight the potential role of these genes in attenuating the toxicity of certain metals and could represent an important means of bacterial resistance against chemicals used to control diseases.

The genus *Xanthomonas* includes pathogenic bacteria that are highly damaging to a number of economically important plant hosts, including citrus species. *Xanthomonas axonopodis* pv. citri is the cause of citrus canker, with most commercial citrus varieties being moderately to highly susceptible to this disease. Typical symptoms of citrus canker are circular, necrotic spots with a water-soaked margin surrounded by a yellow halo on leaves and fruits. Heavily affected plants suffer defoliation, dieback and fruit drop.

The main strategies for controlling citrus canker include quarantine measures to prevent the introduction and establishment of bacteria in orchards, and the use of copper compounds to reduce contamination and dissemination of the disease. The use of copper sprays is a major preventive measure in most integrated management programs. The application of copper to young citrus leaves protects against infection, but this protection is quickly lost because of the fast expansion of the leaf surface area. Indeed, copper compounds are less effective in preventing leaf infection than in preventing fruit infection, primarily because of the slower growth rate of the latter.

Copper induces metabolic changes in several plant-associated bacteria, such as *Rhizobium leguminosarum*, *Agrobacterium tumefaciens*, *Ralstonia solanacearum*, *Xanthomonas campestris* pv. campestris, and *Erwinia amylovora*, as part of the survival strategy of these microorganisms (Alexander *et al.*, 1999; Colwell, 2000; Grey and Steck, 2001a,b: da Silva *et al.*, 2002). *X. axonopodis* pv. citri contains homologues to genes (*copA*, *copB, cutC* and *dsbD*) that are closely related to resistance against toxic levels of copper in other xanthomonads and *Pseudomonas syringae* (Lee *at al.*, 1994; Mellano and Cooksey, 1988; Mills *et al.*, 1993; Voloudakis *et al.*, 2005; Teixeira *et al.*, 2008**)**. Much of the information available on the factors that affect *X. axonopodis* pv. citri and its response to copper has been deduced from other bacteria (Basim *et al.*, 2005; Voloudakis *et al.*, 2005). However, recently, the operon *cop*AB of *X. axonopodis* pv. citri was shown to involved in the copper resistance of this species (Teixeira *et al.*, 2008).

We have previously reported the expression of 279 genes of *X. axonopodis* pv. citri putatively involved in modulating the pathogenicity and virulence of this species, including resistance to toxic compounds (Astua-Monge *et al.*, 2005). To understand the global response of *X. axonopodis* pv. citri to copper, we used real-time quantitative PCR to examine the profile of 32 of the 279 genes putatively associated with pathogenicity and virulence. Almost all of the genes were up-regulated. Two genes homologous to members of the type II secretion system (*xcs*H and *xcs*C) and two involved in the degradation of plant cell wall components (*pgl*A and *pel*) were the genes most expressed in response to an elevated concentration of copper.

To examine the gene expression in response to a high concentration of copper *in vitro*, *X. axonopodis* pv. citri strain 306 was grown overnight in 200 mL of nutrient broth (5 g of peptone/L and 3 g of meat extract/L, pH 7.0) and then centrifuged (10,000 x *g*, 4 °C, 15 min). The bacteria were harvested and resuspended in 285 mL of M9 minimum medium (Na_2_HPO_4_ 6 g/L, KH_2_PO_4_ 3 g/L, NaCl 0.5 g/L and NH_4_Cl 1 g/L) to which 0.2 mM CuSO_4_ was added after filtration through a 0.45 μm filter; this concentration of CuSO_4_ corresponded to the minimum inhibitory concentration (MIC) and was determined by growing bacteria in M9 medium supplemented with 0.04, 0.08, 0.12 and 0.20 mM CuSO_4_. Control cultures contained the same medium but without copper. The cultures were incubated at 28 °C overnight with shaking (100 rpm) and aliquots of 50 mL were harvested after 2, 4, 6, 8, 10 and 12 h, centrifuged and stored at -80 °C. The six samples were subsequently pooled and used for RNA extraction. All of the experiments were done in triplicate.

Total RNA was extracted by lysing the bacterial cells with lysozyme (0.5 mg/mL in Tris-HCl/EDTA buffer, pH 8.0) followed by incubation with 10% SDS at 64 °C and treatment with chloroform. The RNA samples were treated with DNase (*DNase I* Amp Grade, Invitrogen) according to the manufacturers instructions followed by purification with phenol, phenol:chloroform (50:50, v/v) and chloroform. Finally, the RNA samples were precipitated with 3 M sodium acetate and 100% cold ethanol and stored at -80 °C until required. Contamination by DNA was confirmed by PCR using specific primers for the gene avrXacE1 from *X. axonopodis* pv. citri.

cDNA was synthesized in random hexamer-primed reactions from approximately 5 μg of DNase I-treated RNA by single-step reverse transcription RT-PCR done according to the protocol provided by Invitrogen for M-MLV. Briefly, to each RNA sample were added 0.5 μL of random primer (3 μg/μL), 1 μL of 10 mM dNTPs and DEPC-treated water to a final volume of 12 μL. The samples were incubated at 68 °C for 15 min and then immediately placed on ice for 3 min. A second reaction was prepared by using 5X first-strand buffer, 0.1 M DTT, RNase-out and M-MLV reverse transcriptase followed by incubation in a water bath 37 °C for 1 h.

The level of abundance of the pathogenicity-related genes *acvB, avrBs2, avrXacE1, avrXacE2, celD, copA, copB, cutC, egl, gumB, gumG, gumM, hpaA, hrcR, hrcT, hrpB1, hrpG, ostA, pel, pglA, rpfC, rpfE, rpfF, virB8, virD4, virK, xanB, xcsC, xcsH, xpsD, xpsL, and xrvA*, and transcripts over time relative to the *lrp* gene (leucine-responsive regulatory protein gene, used as an internal control) was analyzed by RT-qPCR. Primers for PCR were designed to amplify internal fragments of 100-200 bp in each open reading frame (Table 1) and were tested for amplification specificity and efficiency. Each reaction was done in a total volume of 25 μL that contained 1 μL of cDNA (60 ng/μL), 2.5 mM of each primer and 12.5 μL of SYBR Green PCR Master Mix kit (PE Applied Biosystems, Foster City, CA). The cycling conditions used were 2 min at 50 °C, 10 min at 95 °C and 40 cycles of 30 s at 95 °C and 1 min at 60 °C, with a final extension of 10 min at 72 °C. The cDNA was quantified with an ABI Prism 7500 sequence detection system (PE Applied Biosystems). The comparative cycle threshold method was used to analyze the data, as described by the manufacturer (PE Applied Biosystems). The ΔΔCt method was used to calculate the relative amount of specific RNA present in a sample, from which the fold induction of gene transcription was estimated by comparison to the values for control colonies grown in the same minimal medium but with no addition of copper. Each PCR was done in triplicate and the mean data from 2-3 experimental replicates are reported.

Although a number of *X. axonopodis* pv. citri genes have been implicated in processes related to detoxification (da Silva *et al.*, 2002), little is known about the regulation of the copper stress response in this species. A better understanding of the pathways involved in resistance to copper could be useful in identifying potential target genes for further studies. In this work, we used qPCR to examine the response of *X. axonopodis* pv. citri after exposure to an elevated concentration of copper sulfate for up to 12 h. The genes investigated here were chosen based on the results of a previous study that used DNA macroarrays (Astua-Monge *et al.*, 2005) (Table 1).

The level of gene expression was estimated based on the cycle threshold (Ct) values and the use of standard curves. In all cases, the data were normalized relative to the constitutively expressed *lrp* gene. The *lrp* gene encodes a major transcriptional regulatory protein involved in the control of at least 75 genes in *E. coli* (Newman *et al.*, 1992), and Lrp homologues are involved in the regulation of amino acid metabolism (Brinkman e*t al.,* 2003). This gene was chosen as an internal control because previous experiments had shown that its transcript levels were not significantly altered compared to those of other genes (*pthA, rpo, nuoB, petC* and 16S rRNA). In addition, this gene is universally present in *Xanthomonas* spp. and has been used to assess the phylogenetic relationships within this genus (Cubero and Graham, 2004, 2005).

In the present study, some of the operons chosen for analysis were clusters from which the initial, central and terminal genes were used to monitor transcription. Overall, gene expression in the presence of copper was up-regulated by 0.03-5.72 fold (Table 1).

RT-qPCR indicated that the transcripts of two transporters (*xcsC* and *xcsH*) and a protein involved in the degradation of plant cell wall components (*pel*) were nearly five-fold higher in medium containing copper compared to that without copper. In addition, the expression of *pglA*, another homologous protein involved in the degradation of plant cell wall components, was approximately two-fold higher in the presence of copper. The *copA* and *copB* genes*,* which are directly associated with copper resistance (Russel, 1998), also showed enhanced expression in the presence of copper, but at a lower level than for transporter (*xcs*H and *xcs*C) and exoenzyme (*pgl*A and *pel*) genes.

The homologue genes *xcsH* and *xcsC* (OK) encode putative transport proteins of the type II secretory apparatus involved in the translocation of exoproteins from the cytoplasm to the periplasm. Interestingly, all of the other homologous proteins, *i.e.*, those involved in degrading plant cell wall components, that showed higher expression during copper stress may also be translocated by the type II secretion systems (Russel, 1998; Jha *et al.*, 2005; Johnson *et al.*, 2006).

Although most of the genes examined here were only slightly up-regulated, a set of four genes related to pathogenicity and virulence showed markedly enhanced expression, including two type II secretion system components and two genes involved in degrading components of plant cell walls. These genes are associated with pathogenicity and virulence-related genes of *X. axonopodis* pv. citri identified in hrp-inducing medium (XVM2) (Astua-Monge *et al.*, 2005). Since it is not feasible to reproduce *in vitro* the restrictive environmental conditions required to identify all of the *X. axonopodis* pv. citri genes involved in plant colonization there is a need for individual analysis of the key virulence factors involved in this process.

The type II secretion system is used by various Gram-positive bacteria to transport a large number of secreted proteins, including major virulence factors (such as pectate lyases, cellulases, proteases, toxins and alkaline phosphatases) and other proteins associated with the induction of plant defense responses, from the periplasmic space to the extracellular environment (Russel, 1998; Jha *et al.*, 2005; Johnson *et al.*, 2006). Interestingly, *X. axonopodis* pv. citri contains two copies (*xps* and *xcs* operons) of this machinery, but there is no homology between the two clusters. Although *X. axonopodis* pv. citri harbors two type II secretion systems (*xps* and *xcs*), only *xcs* was expressed to a significant extent. These two systems are probably involved in the secretion of different exoproteins, as in *Pseudomonas aeruginosa*, which has two functional type II systems (Filloux *et al.*, 1998; Ball *et al.*, 2002). Despite the fact that bacterial secretion systems are important for host-pathogen interactions, in many bacteria the type II secretion system is responsible for the transport of a number of virulence-associated proteins (Iwobi *et al.*, 2003; Arrieta *et al.*, 2004; Rossier *et al.*, 2004; Soderberg *et al.*, 2004).

In *X. axonopodis* pv. citri the *copAB* operon is specifically induced by copper (Teixeira *et al.*, 2008). We also observed up-regulation of *cop*A and *cop*B, but to a lower extent than for genes associated with secretion systems. Neither of the transcripts was detected in incubations without copper but showed enhanced expression in the presence of this metal (Table 1). Since *X. axonopodis* pv. citri is copper-sensitive, the apparent difference in the expression of *cop*A and *cop*B may be related to the method of transcript detection (northern blot versus RT-qPCR) or to the activity of copper in the medium. In our experiments, the MIC was estimated in inorganic medium (M9) that lacked chelating agents usually present in organic media such as TSA (1% trypone, 1% sucrose and 0.1% sodium glutamate) or nutrient broth (see Material and Methods). On the other hand, there could be a direct relationship between the external concentration of copper and expression of the *cop*AB operon. Since the MIC values obtained using different culture media cannot be compared, the corresponding levels of gene expression are also not directly comparable. Despite the absence of *copC* and *copD* in the *cop* operon of *X. axonopodis* pv. citri*,* the bacteria were apparently able to detoxify copper at low concentrations that affected growth but did not kill the cells.

Although a lack of growth in the presence of high levels of copper is generally considered to be indicative of cell death, this absence of growth could also result from the cells entering the viable but non-culturable (VBNC) state (Grey and Steck, 2001b). This state is defined as one in which cells are viable but do not undergo sufficient division to allow visible growth in non-selective media (Colwell, 2000). After exposure to a high concentration of copper sulfate, *E. amylovora* cells in the VBNC state regained their ability to grow and their pathogenicity (Ordax *et al.*, 2006). Our results indicate that copper may cause *X. axonopodis* pv. citri to enter the VBNC state.

In contrast to *Cupriavidus metallidurans*, a model organism remarkable for its copper-resistance in which exposure to elevated Cu(II) concentrations results in gene inductions of 2- to 1159-fold (Monchy *et al.*, 2006), the copper-resistance genes of *X. axonopodis* pv citri were only slighly up-regulated by exposure to copper. In *E. amylovora*, another bacterial plant pathogen, the MIC of copper sulfate in solid medium was 3.5 mM Cu^2+^, which was 70 times higher than the highest copper concentration (0.05 mM Cu^2+^) used in the survival experiments (Ordax *et al.*, 2006). In *Xanthomonas campestris* pv. juglandis, the expression of all four ORFs (*copABCD)* that showed homology with the operons for tolerance against copper was increased following exposure to copper; this enhanced expression was required for full resistance to this metal (Lee *et al.*, 1994).

In some organisms, the *cop* operon is induced by copper, which allows growth in the presence of high levels of copper compounds (up to 5 mM) used for disease control in agricultural crops. In *C. metallidurans*, a bacterial species with a notorious resistance to metals, resistance to copper is represented by two gene clusters that putatively encode periplasmic resistance (*copSRABCD* and *copVTMKNSRABCDIJGFLQHE*), with maximal expression at 0.4 mM Cu (Monchy *et al.*, 2006).

In conclusion, the results described here indicate that exposure to copper induces the differential expression of several pathogenicity-related genes in *X. axonopodis* pv. citri. The most highly induced genes (such as the *xcs* operon, *pel* and *pglA*) are related to high levels of copper and transport, whereas the genes involved in other activities showed a lower level of induction. The responsive genes *xcs*C and *xcs*H are members of the type II secretion system, which confirms the importance of this system in the response to copper compounds in *X. axonopodis* pv. citri. Whether the enhanced expression of these genes in response to a high copper concentration is associated with a mechanism of detoxification or is simply a response to the presence of copper as a co-factor remains to be determined. The identification of a type II secretion system as a component involved in the response of *X. axonopodis* pv. citri is an essential step towards a more comprehensive understanding of the role of these genes in bacterial adaptation to copper-induced stress.

## Figures and Tables

**Table 1:** ORF names, primers for quantitative PCR (qPCR) and transcript levels of pathogenicity and virulence genes of *Xanthomonas axonopodis* pv. citri grown under copper stress.

ORF name	Protein name Forward (F) and reverse (R) primers	Fold increase ± SD^a^
*acvB*	Virulence protein F – tgcaactggtccatgatcg R – gacggtgtgcatctacgg	0.29 ± 0.05
*avrBs2*	Avirulence protein F – agacaacgcgatcacacc R – caatccgtctccgtctgc	0.79 ± 0.22
*avrXacE1*	Avirulence protein F – acagcgatcctgaaagacg R – cttcgataccagaaagcctgc	0.37 ± 0.25
*avrXacE2*	Avirulence protein F – ctgaggaagtctggcaacc R – cgcttgctgctttcttgc	0.40 ± 0.26
*celD*	Glucan 1,4-beta-glucosidase F – gatgtgaccaagctgaccc R – tacttgaccgggcagtcg	0.07 ± 0.06
*copA*	Copper resistance protein A precursor F – aacttccaggtacgcaagc r- catgtggtacagcagatggc	0.10 ± 0.06
*copB*	Copper resistance protein B precursor F – ggcgaatacgaggtactgc R – gcatccagccgatgtacg	0.46 ± 0.08
*cutC*	Copper homeostasis protein F – tttccgagcacaacatcc R – gtccaccatccgttgtacc	0.07 ± 0.01
*Egl*	Cellulase F – atggaaaagaacagcgacg R – cgtgcgtacttgctcagg	0.18 ± 0.02
*gumB*	GumB protein F – atcctgagatctatggcgg R – gccacaccatcacaagagg	0.21 ± 0.00
*gumG*	GumG protein F – gattctgtgcacgcatacc R – catttgcgtttcaacccc	0.46 ± 0.03
*gumM*	GumM protein F – gcatatggaatggatgtatcg R – caggtgcggaagaacacc	0.08 ± 0.08
*hpaA*	Type III secretion system protein F – acgcaattttcacctattgc R – ctcaatcagtagtgtgttcagacg	0.35 ± 0.19
*hrcR*	Type III secretion system protein R F – ggtgtttatcgtcatcgacc R – accgatagctcagaaccagg	1.83 ± 0.23
*hrcT*	Type III secretion system protein T F – aatttgagtcagccaatccg R – caattgctgctggaaatgc	0.61 ± 0.24
*hrpB1*	Type III secretion system protein B1 F – gctgatcgaagaagacgc R – caggcacgcaggtattgc	0.79 ± 0.10
*hrpG*	Type III secretion system protein G F – cagcacatctacaagttgcg R – ccttgctcattgtcgttgc	0.21 ± 0.04
*ostA*	Trehalose-6-phosphate synthase F – gaaatgaaggaagcgttgc R – agagagcgcttgcagatagc	0.19 ± 0.03
*pel*	Pectate lyase F – caacgtgttcgagatcagc R – tttcgccttgacgtctgc	5.29 ± 0.72
*pglA*	Polygalacturonase F – acttcaccttcaagggtggc R – cggcagtaccgagtgatagg	1.89 ± 0.28
*rpfC*	Regulation pathogenicity factor C F – acagttgaagaccggactgg R – tcagggatcgccattacc	0.26 ± 0.24
*rpfE*	Regulatory protein F – tggtgttggacttccagg R – cattgcgccagctgtacc	0.13 ± 0.04
*rpfF*	Regulation pathogenicity factor F F – gtacctggccatgaatgc R – gcagcgacttttcattgagc	0.14 ± 0.02
*virB8*	Type IV system protein B8 F – atttcctgatcaaatgcagc R – tcatcgaccacttgctcc	0.37 ± 0.10
*virD4*	Type IV system protein D4 F – catagcgtgtcagaagaacg R – gatggtaagcgtcttcacttcg	0.09 ± 0.01
*virK*	VirK protein F – agcagttcatgcgctacc R – gcattgatcttgcattgatacg	0.19 ± 0.04
*xanB*	Phosphomannose isomerase/ GDP-mannose pyrophosphorylase F – aaccagagcacctacatccc R – ataatcgtcttcgcccagatagc	0.04 ± 0.03
*xcsC*	Type II secretion system protein C F – gctgacccagatcaatgg R – gcttcaaggtcatggtgtgg	4.72 ± 0.64
*xcsH*	Type II secretion system protein H F – atcgccttggatgaaacg R – catcgacctgcactacgc	5.72 ± 0.15
*xpsD*	General secretion pathway protein G F – ccttcaattgtgcgtaatcc R – attttccatcccgacttatcg	0.03 ± 0.01
*xpsL*	General secretion pathway protein L F – cctggtggtacgtatctgg R – gtgatggtgaagcggtcg	1.17 ± 1.12
*xrvA*	Virulence regulator F – aaggaagtggtcaagagcg R – aaccagcggaaacttgagg	0.05 ± 0.01

^a^Fold increase in the level of gene expression compared to the control ± standard deviation (SD).
